# Evaluation of Bioactive Properties of Ultrasound-Assisted Extracts from Prokupac Grape Skins for Functional Foods

**DOI:** 10.3390/antiox14060733

**Published:** 2025-06-15

**Authors:** Edina Avdović, Dušan Dimić, Đura Nakarada, Dušica Simijonović, Sandra Jovičić Milić, Katarina Marković, Mirjana Grujović, Marko Antonijević, Andrija Ćirić, Dejan Milenković, Zoran Marković

**Affiliations:** 1 Institute for Information Technologies, University of Kragujevac, Jovana Cvijića bb, 34000 Kragujevac, Serbiasandra.jovicic@pmf.kg.ac.rs (S.J.M.); katarina.mladenovic@pmf.kg.ac.rs (K.M.); mirjana.grujovic@pmf.kg.ac.rs (M.G.); mantonijevic@uni.kg.ac.rs (M.A.); dejanm@uni.kg.ac.rs (D.M.); 2 Faculty of Physical Chemistry, University of Belgrade, Studentski trg 12–16, 11000 Belgrade, Serbia; djura@ffh.bg.ac.rs; 3 Faculty of Science, University of Kragujevac, Radoja Domanovića 12, 34000 Kragujevac, Serbia; andrija.ciric@pmf.kg.ac.rs; 4 Department of Natural Sciences and Mathematics, State University of Novi Pazar, Vuka Karadžića bb, 36300 Novi Pazar, Serbia

**Keywords:** circular economy, Prokupac grape skin extracts, HPLC analysis, antioxidant and anti-inflammatory potential, EPR, antimicrobial activity

## Abstract

The phenolic compounds present in wine industry by-products are a valuable source of biologically active ingredients that could be used in the development of functional foods. This manuscript investigates the potential of ultrasound-assisted extracts from Prokupac grape skins—a wine industry by-product—as functional food ingredients. Four extracts were prepared using different solvents and evaluated for their antioxidant, anti-inflammatory, and antimicrobial properties. Antioxidant activity was assessed through DPPH, ABTS, and FRAP assays, as well as EPR spectroscopy. Phenolic composition was determined via HPLC analysis, and anti-inflammatory potential was evaluated using a lipoxygenase inhibition assay. Results indicated that the extracts PSE3 (ethyl acetate) and PSE0 (direct extraction with 50% ethanol) exhibited superior antioxidant and anti-inflammatory activities, which can be attributed to their high polyphenolic content. Additionally, the extracts demonstrated antimicrobial effects against the tested microorganisms. These findings suggest that Prokupac grape skin extracts, particularly PSE3 and PSE0, could be valuable additions to functional foods, offering health benefits through their bioactive properties.

## 1. Introduction

The wine industry generates a large amount of waste, mainly consisting of grape skins and seeds [1]. The reprocessing of waste reduces its negative impact and yields high value-added materials [2]. Some residues are used as organic fertilizers and food for animals. The high antioxidant potential of red wine originates from polyphenolic compounds. Various biologically active compounds—including epicatechin, procyanidin B, quercetin glucoside, and astringin—are characteristic of different varieties [3]. Morre and Morre previously posited that grape skin is a much better source of bioactive compounds than grape pulp, juice, and seeds [4]. The aforementioned chemicals are recognized as potent radical scavengers, as experimentally shown towards model (DPPH and ABTS) and biologically relevant (hydroxyl, ascorbyl, and hydroperoxyl) radicals [5,6,7]. Also, several studies have shown an inverse correlation between phenolic content and in vitro oxidation of LDL [8]. Moura et al. proved that the grape skin extracts (GSEs) reduced the systolic, mean, and diastolic arterial pressure of Wistar rats, along with the concentration-dependent inhibition of malonaldehyde production, which served as a marker for lipid peroxidation [9]. The antimicrobial activity of GSEs toward several Gram-positive and Gram-negative bacteria has been proven in several references, such as Katalinić et al. (2010) [3]. The anticancer activity of these extracts toward prostate, breast, colorectal, and skin cancers have also been reported [10,11,12,13].

The extraction of phenolic compounds from grape skins can be carried out using several methods. The most common techniques include solid–liquid extraction and Soxhlet extraction with solvents like ethanol and methanol [14,15]. Due to the long extraction times often associated with these methods, oxidation and hydrolysis of biologically active compounds can occur [16]. Therefore, there is an increasing number of publications in which ultrasound- and microwave-assisted extractions are applied to wine industry waste to shorten extraction time and increase phenolic content [17,18]. Ultrasound-assisted extraction is based on cavitation phenomena and the implosion of air bubbles in liquids. This generates high temperature and pressure due to the compression of gases, which stimulates solvent penetration in the solid phase, thereby enhancing the removal of soluble compounds [2].

The Šumadija region of Serbia, known for its rich viticultural heritage, boasts the ideal climate and soil composition for high-quality grape production [19]. The combination of moderate continental climate, well-drained soils, and traditional winemaking techniques contributes to cultivating grape varieties rich in bioactive compounds [20,21]. These grapes, particularly their skins, are abundant in polyphenols, flavonoids, and anthocyanins, which exhibit potent antioxidant and anti-inflammatory properties [21]. Radovanović et al. showed that the concentration of total anthocyanins detected at 520 nm was the highest in wine from this region compared to other Balkan regions [22]. Extracts derived from Šumadija grape skins offer several potential health benefits, including protection against oxidative stress-related diseases and inflammation, further highlighting the region’s significance in viticulture and functional food research [23,24].

Given the growing interest in sustainable sources of bioactive compounds, grape skins represent a promising raw material for functional applications due to their rich phenolic content and biological activity.

This study aims to evaluate the antioxidant, anti-inflammatory, and antimicrobial potential of ultrasound-assisted extracts from Prokupac grape skins, emphasizing their phenolic composition and potential application in functional food development.

## 2. Materials and Methods

### 2.1. Reagents and Chemicals

The chemicals used in this study, including 1,1-diphenyl-2-picrylhydrazyl radical, potassium persulfate, 2,2′-azino-bis(3-ethylbenzothiazoline-6-sulfonic acid) diammonium salt, trichloroacetic acid, potassium ferricyanide, ferric chloride, the referent standards (galic acid, 3,5-dihydroxybenzoic acid, 3,4-dihydroxybenzoic acid, 4-hydroxybenzoic acid, chlorogenic acid, caffeic acid, catehin, syringic acid, epicatehin, ferulic acid, sinapic acid, p-coumaric acid, rutin, myricetin, naringin, quercetin, apigenin, and naringenin), and methanol were procured from Sigma-Aldrich (Steineheim, Germany). Ethyl acetate, ethanol, methanol, acetonitrile, and formic acid (HPLC grade) were purchased from Merck^®^ (KGaA, Darmstadt, Germany). Dimethyl sulfoxide (DMSO) was purchased from Acros Organics (Fairlawn, NJ, USA). Doxycycline was purchased from Sigma Chemicals Co. (St Louis, MO, USA), while itraconazole was purchased from Pfizer (New York, NY, USA). Standard stock solutions for HPLC examination were prepared in methanol (1 mg/mL), stored in the refrigerator, and used within one week. The UV–Vis determinations were performed on a Perkin Elmer Lambda 365 UV/Vis Spectrophotometer (Waltham, MA, USA).

### 2.2. Samples of Grape Skin

Grape skin samples for extraction were obtained from Miletić Winery, situated in the Šumadija region of Serbia. The grape skins were a by-product of rosé wine production, derived from Prokupac, an indigenous Serbian red grape variety. Prokupac is noted for its thick skin, high sugar content, and richness in phenolic compounds, making it suitable for extraction.

After harvesting, the skins were thoroughly washed with distilled water to remove impurities. Drying was performed in a dark, well-ventilated room at ambient temperature (22–24 °C) for seven days, until constant weight was achieved. The dried material was then stored in airtight, light-protected containers at 5 °C until further use in the extraction process.

### 2.3. Ultrasound-Assisted Extraction (UAE)

Ultrasound-assisted extraction (UAE) is an efficient technique that minimizes or eliminates the use of organic solvents while maximizing extraction efficiency. This method has previously been applied to extract polyphenolic compounds from black tea, grape seeds, and various food by-products [20,25,26,27,28].

In this study, grape skins were collected, thoroughly washed, and air-dried. A 20 g portion of each sample was dissolved in 200 mL of three different solvents—ethyl acetate, absolute ethanol, and 50% ethanol. The extraction process was carried out using an ultrasonic bath set at 40 kHz and 150 W for 30 min at 40 °C. During this process, phenolic compounds were released into the solvent. Following extraction, the mixtures were centrifuged at 2490× *g*-force for 10 min to separate the plant matrix from the extracts. The supernatant was collected and filtered using a PTFE syringe filter (0.45 µm). The solvent was removed using a rotary evaporator at 40 °C under reduced pressure. Finally, the extracts were stored at 5 °C. All extractions were performed in triplicate, and the yield percentages are presented in a later section of this paper.

The extracts were labeled as follows: Prokupac grape skin extract in 50% ethanol (PSE1), Prokupac grape skin extract in absolute ethanol (PSE2), Prokupac grape skin extract in ethyl acetate (PSE3), and Prokupac grape skin extract without prior maceration (PSE0).

### 2.4. Determination of Total Phenolic Content

The total phenolic content (TPC) of the extracts was determined using the Folin–Ciocalteu method, following established protocols [29,30,31]. Within this approach, 50 µL of the extract solution of various concentrations was mixed with 250 µL of Folin–Ciocalteu reagent (diluted 1:2 with distilled water) in a test tube. After allowing the mixture to stand at room temperature for 5 min, 750 µL of 20% sodium carbonate (Na_2_CO_3_) solution was added, and the final volume was adjusted to 5 mL with deionized water. The samples were incubated in the dark for 90 min. The absorbance of the samples was determined at 765 nm using a spectrophotometer. TPC values were expressed as milligrams of gallic acid equivalents (GAE) per milligram of dry grape skin extract (GSE) (mg GAE/g GSE). Each sample was analyzed in triplicate, and the results are reported as the mean ± standard deviation.

### 2.5. In Vitro Antioxidant Potential Assessment

Three standard methods were employed to evaluate the antioxidant activity of grape skin extracts used in this study. These included two assays for reducing stable radicals (DPPH and ABTS) and one for reducing Fe^3+^ to Fe^2+^ ions. These methods are widely used due to their standardization, cost-effectiveness, and applicability to a broad range of samples.

#### 2.5.1. DPPH Radical Scavenging Assay

The DPPH radical scavenging assay was used to assess the radical-reducing ability of grape skin extracts, following established protocols [32,33,34]. These extracts were dissolved in methanol in various concentrations. A stock solution of DPPH (0.05 mM) was prepared in methanol and stored in the dark to prevent degradation. Samples of different concentrations of skin extracts were mixed with the same amount of DPPH solution and incubated at 25 °C for 30 min. The control sample contained only DPPH, which was the same amount used for the measurements. The UV–Vis spectrum’s absorbance at 517 nm was used to measure the quantity of DPPH radicals. The inhibitory concentration (SC_50_) was calculated as the amount of sample required to reduce 50% of the DPPH radicals. The measurements were repeated three times, and the results are presented as mean values ± standard deviation. This procedure ensured the reproducibility and reliability of the measurements. Standard antioxidant compounds (nordihydroguaiaretic acid (NDGA), ascorbic acid, and quercetin) were used as positive controls.

#### 2.5.2. ABTS Radical Cation Decolorization Assay

The ABTS test—another widely used method for assessing the antioxidant capacity of samples—was also included in this study. The ABTS radical cation (ABTS^•+^) was generated in the reaction between 2,2′-azino-bis(3-ethylbenzothiazoline-6-sulfonic acid (ABTS) and potassium persulfate [32,33,34]. The mixture was kept in the dark for 16 h to ensure complete radical formation. The stock solution of ABTS^•+^ was prepared in methanol, adjusted to an absorbance of 0.70 at 734 nm. Meanwhile, the grape skin extracts were dissolved in methanol at varying concentrations.

The procedure also included mixing 1000 μL of diluted ABTS^•+^ solution with an equal volume of sample solution. After one minute, the absorbance was measured, and the decrease in its value reflected the amount of scavenged radical. The antioxidant potency of samples is presented as an SC_50_ value.

#### 2.5.3. Ferric Reducing Antioxidant Power (FRAP) Assay

The FRAP assay is typically used to measure the reducing power of samples in a system containing Fe^3+^ ions, according to references [32,33,34]. In the presence of polyphenolic compounds, these ions are reduced to Fe^2+^ through electron transfer. The stock solutions of the samples were prepared in phosphate buffer at pH = 7.4. The measurements were performed on solutions obtained by mixing 500 μL of diluted extract and 250 μL of 1% potassium ferricyanide. This mixture was incubated at 50 °C for 20 min and later combined with 500 μL of 10% trichloroacetic acid, 100 μL of 0.1% ferric chloride, and 500 μL of deionized water. Upon incubation for an additional 10 min, the absorbance of the solution was measured at 700 nm. The results were compared to those obtained using ascorbic acid as a reference standard.

#### 2.5.4. Measurement of Hydroxyl Radical Scavenging Potential Using Electron Paramagnetic Resonance (EPR) Spectroscopy

Hydroxyl radical scavenging activity was assessed using electron paramagnetic resonance (EPR) spectroscopy [35,36] at room temperature (293 K) on a Bruker Biospin Elexsys II 540 spectrometer (9.65 GHz). The device was run with a sweep time of 60 s, a modulation frequency of 100 kHz, a modulation amplitude of 2 mT, and a power attenuation of 13 dB. Hydroxyl radicals (HO^•^) were generated through a standard Fenton reaction. The reaction mixture, with a total volume of 29 µL, consisted of 2 µL H_2_O_2_ (final concentration 0.35 mM), 1 µL DEPMPO (final concentration 3.5 mM), and 1 µL of 10 mg/mL extract solution (dissolved in water), and the rest was deionized water. EPR spectra were recorded two minutes after introducing 1 µL of FeSO_4_ (final concentration 0.15 mM) into the system. A blank sample, containing the same volume of water in place of the extracts, was used for reference. All measurements were repeated three times, and the standard deviations were calculated.

The scavenging efficiency of the extracts was determined by measuring the decrease in the intensity of the most prominent DEPMPO–HO^•^ adduct peak in the low-field region of the EPR spectrum. The percentage of radical reduction (%*RR*) was calculated using the following formula:

$$\%RR=\frac{I_{0}-I_{a}}{I_{C}}100$$

where *I*_0_ represents the peak intensity of the DEPMPO/HO^•^ adduct in the absence of extracts, and *I*_a_ represents the intensity in their presence.

#### 2.5.5. Measurement of Ascorbyl Radical Scavenging Potential Using Electron Paramagnetic Resonance (EPR) Spectroscopy

To determine the scavenging potential of the extracts toward ascorbyl radicals, the EPR signal of ascorbyl radicals (Asc^•^) in DMSO (dimethyl sulfoxide) solution was recorded in a system containing extracts in DMSO, using the previously developed procedure [37,38]. Briefly, 10 µL of extracts in DMSO, 10 µL of aqueous ethylenediaminetetraacetic acid (EDTA) solution (final concentration 0.2 mM), and 1 µL of aqueous FeCl_3_ solution (final concentration 7 µM) were mixed to form the Fe(III)-EDTA complex, followed by the addition of 69 µL of DMSO. Then, 10 µL of DMSO solution of ascorbic acid (final concentration 0.2 mM) was added to the mixture, and 30 µL was transferred into the gas-permeable Teflon tube. This tube was placed into a quartz EPR cuvette, which was inserted into an EPR resonator, and the EPR signal was recorded after 2 min using the following experimental parameters: center field 3500 G, microwave power 10 mW, microwave frequency 9.85 GHz, modulation frequency 100 kHz, and modulation amplitude 1 G. Control recordings were obtained by adding 10 µL of DMSO instead of the extracts. The antioxidant activity of the extracts was calculated using the previously described formula. All measurements were repeated three times, and the standard deviations were calculated.

### 2.6. HPLC Analysis

The phenolic content of the extracts was analyzed using high-performance liquid chromatography (HPLC) on a Shimadzu Prominence HPLC system (Kyoto, Japan) equipped with a photodiode array (PDA) detector (SPD-M20A). Separation was achieved using a Hypersil Gold aQ C18 column (Thermo Fisher Scientific, Waltham, MA, USA) (150 × 4.6 mm, 5 µm). Two solvents were used as the mobile phase: (A) 0.1% formic acid in deionized water and (B) 0.1% formic acid in acetonitrile. A gradient elution program was employed with the following profile: 2% B at 0–2 min, increased to 95% B over 45 min. The flow rate was 1 mL/min, and the injection volume was 20 µL. The quantification of compounds was conducted by measuring the absorption spectra at wavelengths specific for selected compounds, for example, 280 nm for phenolic acids and flavonoids [29,30]. The identification of compounds was based on comparing retention times and on the shape of the UV–Vis spectra of standards, as explained in [33,34]. Shimadzu LabSolutions software was used for the quantification of compounds. All experiments were performed in triplicate to ensure reproducibility and accuracy.

### 2.7. Lipoxygenase (LOX) Inhibition Assay

Lipoxygenase (LOX) is an enzyme that catalyzes the oxidation of polyunsaturated fatty acids, leading to the generation of hydroperoxides, which serve as precursors to biologically active molecules such as leukotrienes. These compounds are crucial in inflammatory processes, particularly allergic reactions, asthma, and chronic inflammatory disorders. Prolonged inflammation leads to sustained oxidative stress and an excessive release of inflammatory mediators, including cytokines, prostaglandins, and leukotrienes, which can contribute to tissue damage and disease progression. Since LOX activity is closely linked to inflammatory pathways, its inhibition can effectively reduce the production of pro-inflammatory molecules, thereby alleviating symptoms and potentially preventing complications associated with chronic inflammation. As a result, LOX inhibitors have gained attention in therapeutic research for their potential in treating inflammatory and immune-related disorders.

The anti-inflammatory activity of extracts was determined using the LOX inhibition assay [39,40,41]. A solution of soybean lipoxygenase was prepared in saline. The sodium linoleate substrate solution was then mixed with a Tris buffer (pH = 9). The grape skin extracts were dissolved in DMSO and diluted to various concentrations. The measured samples contained the extract, Tris buffer, LOX enzyme solution (with a final concentration of 5 × 10^3^ units/mL), and sodium linoleate (with a final concentration of 3 mM). LOX activity was determined by measuring the absorbance at 234 nm, corresponding to the concentration of conjugated dienes. NDGA was used as a positive control. The activity was expressed as the IC_50_ value—the concentration required to inhibit 50% of the LOX activity [42]. The measurements were performed in triplicate, and the IC_50_ values are presented as mean ± standard deviation.

### 2.8. In Vitro Antimicrobial Assay

#### 2.8.1. Microorganisms Used for Testing

The antimicrobial activity of the tested extracts was evaluated against 12 microorganisms. Gram-positive bacteria included *Bacillus subtilis* and *Staphylococcus aureus*, along with their respective ATCC strains (*B. subtilis* ATCC 6633 and *S. aureus* ATCC 25923). In contrast, Gram-negative bacteria included *Proteus mirabilis*, *Pseudomonas aeruginosa*, and *Escherichia coli*, along with their ATCC strains (*P. mirabilis* ATCC 12453, *P. aeruginosa* ATCC 27853, and *E. coli* ATCC 25922). Lastly, yeasts included *Candida albicans* and its reference strain *C. albicans* ATCC 10231.

This selection was guided by the clinical and food-related significance of these microorganisms and their distinct cell wall structures, which affect their susceptibility to antimicrobial agents. The inclusion of both Gram-positive and Gram-negative bacteria enabled a comprehensive evaluation of the antibacterial spectrum of the extracts, while *C. albicans* served to assess antifungal potential. The use of both wild-type and ATCC reference strains further enhances the relevance of the findings.

#### 2.8.2. Preparation of Suspensions

Bacterial suspensions were prepared following the direct colony method [43]. The turbidity of the initial suspension was adjusted using a densitometer (DEN-1, BioSan, Rīga, Latvia). Once the suspension was adjusted to match the turbidity of a 0.5 McFarland standard, it contained approximately 108 colony-forming units (CFU) per milliliter for bacteria and about 106 CFU/mL for yeast. Additional 1:100 dilutions of each of the initial suspensions were prepared in sterile 0.85% saline for the experiment.

#### 2.8.3. Microdilution Method

Antimicrobial activity was evaluated by determining the minimum inhibitory concentration (MIC) and minimum microbicidal concentration (MMC) using the microdilution plate method, with resazurin as an indicator [44]. In 96-well plates, 100 μL of Mueller–Hinton broth (Torlak, Belgrade, Serbia) for bacterial testing and Sabouraud broth (Torlak, Belgrade, Serbia) for fungal testing were dispensed into each well. The extracts under investigation were dissolved in a 10% solution of DMSO, followed by dilution in sterile distilled water. A 100 μL aliquot of the stock solution of the tested extract was added to the first row, and twofold serial dilutions were then carried out using a multichannel pipette. The resulting concentration range was from 20 to 0.156 mg/mL. Details of this method are further elaborated in the reference study [33,45].

An antibiotic, doxycycline (Sigma Chemicals Co., Saint Louis, MO, USA), was dissolved in a nutrient liquid medium, Mueller–Hinton broth (Torlak, Belgrade, Serbia). The antifungal agent itraconazole (Pfizer, New York, NY, USA) was dissolved in Sabouraud broth (Torlak, Belgrade, Serbia). Doxycycline is a broad-spectrum antibiotic effective against aerobic and anaerobic Gram-positive and Gram-negative bacteria. Ampicillin, a beta-lactam antibiotic, primarily targets Gram-positive bacteria, as well as some Gram-negative strains. Itraconazole is an antifungal agent used to inhibit the growth of the *Candida* species. The solvent control test with 10% DMSO did not exhibit any inhibition of microbial growth.

Each test also included growth control and sterility control. All tests were conducted in duplicate, and the MIC values were consistent. To determine the minimum microbicidal concentration (MMC), 10 μL of each sample from the wells showing no color change or no fungal growth were plated on nutrient agar. After the incubation period, the lowest concentration at which no microbial growth was observed (i.e., no colonies) was recorded as the minimum microbicidal concentration.

### 2.9. Statistical Analysis

Statistical analysis and comparison of the extracts’ antioxidant activities/phenolic content were assessed using the Statistical Package for the Social Sciences (SPSS) 24.0 software package [46]. The results are presented as the mean ± standard deviation (SD), which were analyzed by the one-way analysis of variance (ANOVA). When statistical differences were observed, the variables were compared using Tukey’s multiple range test. A significance level of 5.0% was selected for the statistical tests.

## 3. Results and Discussion

### 3.1. Extraction and Extraction Optimization

In this study, bioactive phenolic compounds were extracted from the native grape variety Prokupac using UAE. A previous study [33] provided a detailed optimization process for this extraction method. To ensure an eco-friendly approach, solvents such as ethyl acetate, 50% ethanol, and absolute ethanol were used. Extraction conditions were carefully optimized to maximize yield while preserving thermolabile compounds. Following the optimization process, four extracts from the indigenous Prokupac grape variety were obtained and analyzed using HPLC for their antibacterial, anti-inflammatory, and antioxidant properties, as well as their phenolic composition.

The extraction yield varied from 4.9% to 9.6%, depending on solvent polarity (Table 1). The highest yields were obtained from extracts containing 50% ethanol (PSE0 and PSE1), while the ethyl acetate extract (PSE3) had the lowest yield.

### 3.2. Spectrophotometric Determination of the Total Phenolic Content

The TPC values are presented as milligrams of gallic acid equivalents per gram of dry extracts (Table 1). This analysis showed a wide range of values among the extracts studied. The highest phenolic content was present in PSE0 (22.4 mg GAE/g GSE), leading to the expectation that this extract would show the most prominent radical scavenging activity. The extract obtained in ethyl acetate had the second highest phenolic content of 9.8 mg GAE/g GSE. The other two extracts in 50% ethanol and absolute ethanol were characterized by TPC values of 3.6 and 2.2 mg GAE/g GSE. These extracts contained a high amount of phenolic compounds, making them suitable candidates for further studies.

### 3.3. UV–Vis and EPR Determination of Antioxidant Activity

Three standard methods—DPPH, ABTS, and FRAP—were used to evaluate the antioxidant activity of the extracts. Although DPPH^●^ and ABTS^●+^ are not biologically relevant, these methods are standardized and commonly used for assessing antioxidant potential. The combination of these three assays provides a more comprehensive assessment of the antioxidant potential of the tested extracts by targeting different mechanisms underlying antioxidant activity, including radical scavenging and reducing activity.

#### 3.3.1. DPPH Radical Scavenging Activity

Among the tested samples, PSE0 (0.3 µg/mL) and PSE3 (8.3 µg/mL) exhibited the lowest SC_50_ values, indicating strong antioxidant activity. In contrast, PSE1 and PSE2 displayed significantly higher SC_50_ values of 41.9 µg/mL and 99.4 µg/mL, respectively. These findings highlight the crucial relationship between phenolic content and solvent polarity and thus the antioxidant capacity of grape skin extracts. Ethyl acetate was shown to be an effective solvent for extracting polyphenols.

#### 3.3.2. ABTS Radical Scavenging Activity

The ABTS^●+^ reduction assay results align well with those for the DPPH^●^ assay; in some cases, the SC_50_ values are almost identical. This can be seen in the case of PSE0 (0.3 µg/mL) and PSE3 (8.8 µg/mL). Samples PSE1 and PSE2 have a much lower activity, which is consistent with the trend observed in the DPPH^●^ assay. Since ethyl acetate effectively dissolves the components with the highest antioxidant activity, the choice of extraction solvent also plays a crucial role in the ABTS^●+^ assay. To further quantify the specific polyphenol content in each sample, HPLC analysis was performed, as described in the following section.

#### 3.3.3. Ferric Reduction Antioxidant Potential

The ability of the extracts to reduce Fe^3+^ to Fe^2+^ was evaluated using the FRAP assay. A higher absorbance at 700 nm (A_700_) indicates greater iron-reducing potential. Among the tested extracts, PSE0 exhibited the highest A_700_ value (0.2624), aligning well with previous assays described in Section 3.3.1 and Section 3.3.2, where electron donation played a key role in free radical reduction. PSE3 followed with an A_700_ value of 0.1636, while PSE1 and PSE2 demonstrated the lowest reducing potential. When compared to the standard antioxidant ascorbic acid (A_700_ = 0.1249), PSE0 displayed exceptionally high reducing power, whereas the electron-donating ability of PSE3 was comparable to ascorbic acid. The reducing potential of PSE1 and PSE2 was slightly lower than that of the standard antioxidant.

A one-way ANOVA was performed to compare the means of the antioxidant capacities (DPPH, ABTS, and FRAP) across the different extracts (PSE0 to PSE3). The ANOVA results (Table S1) revealed no statistically significant differences between the extracts in the DPPH, ABTS, and FRAP values. The *p*-value (Sig.) of 0.155 is greater than the conventional alpha level of 0.05, indicating that the differences in antioxidant activity among the extracts are not statistically significant. Analysis of the extracts showed that the mean concentrations of antioxidant activity varied widely, with PSEO and PSE3 exhibiting the highest antioxidant activities, particularly in the DPPH and ABTS tests. 

#### 3.3.4. Hydroxyl Radical Reduction Antioxidant Potential

**Figure 1:**
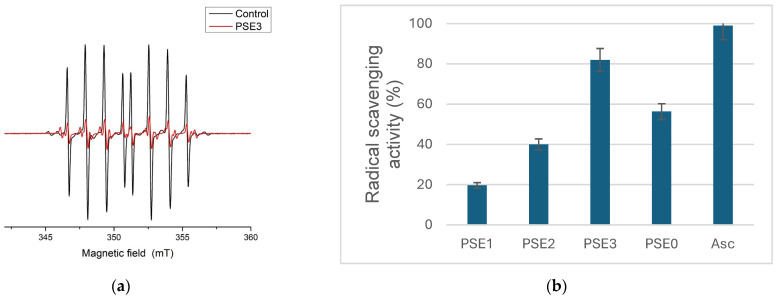
(**a**) Control EPR spectra of DEPMPO/HO^•^ adduct (black) and same representative system containing extract of PSE3; (**b**) antiradical activity of the extracts towards hydroxyl radicals.

Electron paramagnetic resonance (EPR) spectroscopy was used to assess the hydroxyl radical (HO^•^) scavenging capacity of the grape extracts (Figure 1a). Figure 1b illustrates their capacity to scavenge the hydroxyl radical signal.

PSE3 had the highest hydroxyl radical scavenging potential (81.94%) among the investigated samples, followed by PSE0 (56.32%). The significant activity observed for PSE0 further highlights its potential as an important antioxidant source, along with PSE3. In contrast, PSE1 exhibited the lowest scavenging capacity (19.66%), while PSE2 showed moderate activity (39.94%). The varying polarity of the solvents and, therefore, the composition of phenolic and non-phenolic antioxidants in each sample may be responsible for these variations in activity.

#### 3.3.5. Ascorbyl Radical Reduction Antioxidant Potential

**Figure 2:**
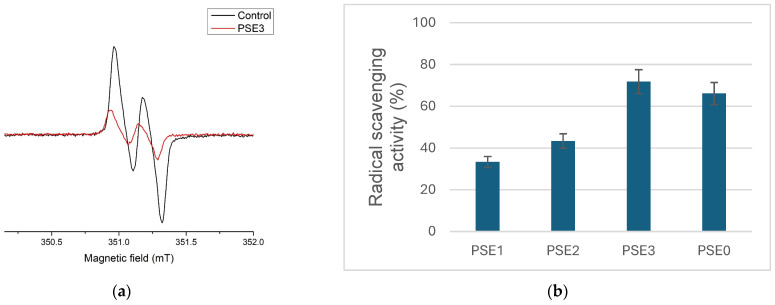
(**a**) Control EPR spectra of ascorbyl radical (black) and same representative system containing PSE3 extract; (**b**) antiradical activity of extracts toward ascorbyl radicals.

The scavenging potential of the grape samples toward ascorbyl radicals was evaluated using electron paramagnetic resonance spectroscopy (Figure 2a), and the results are expressed as the percentage inhibition of the ascorbyl radical signal (Figure 2b).

In alignment with the results of the hydroxyl radical scavenging tests, PSE3 exhibited the highest ascorbyl radical scavenging capacity (71.79%), followed by PSE0 (66.10%), further indicating that these extracts potentially contain high levels of bioactive antioxidants.

PSE1 had the lowest ascorbyl radical scavenging efficiency (33.31%) and the weakest activity toward hydroxyl radicals (19.66%), indicating a weaker antioxidant capability in this sample. Likewise, PSE2 exhibited considerable ascorbyl radical scavenging activity (43.35%) and moderate hydroxyl radical scavenging (39.94%). These findings suggest that the extracts’ capacities to neutralize various radical species may be related, most likely due to their polyphenolic content.

#### 3.3.6. Statistical Analysis of Different Antioxidant Potential Assays

A one-way ANOVA was performed on the sets of radical scavenging activities toward different radicals. As expected, no statistically significant differences were found based on the DPPH, ABTS, and FRAP assays, with a *p*-value between 0.80 and 0.66, all well above the 0.05 significance level. These three tests provided the same order of radical scavenging potency, decreasing in the following order: PSE0 > PSE3 > PSE1 > PSE2. When EPR measurements are considered, there is a deviation in the antioxidant activity order, with PSE3 being the most potent scavenger. The same statistical test was applied to DPPH/OH radical scavenging activity datasets. This analysis showed no statistically significant difference between the two sets, with a *p*-value of 0.976. The differences in the order of the compounds can be a consequence of the radical used. As previously explained, DPPH and ABTS radicals are bulky, with well-protected atoms and unpaired electrons, resulting in their stability. On the other hand, OH radicals are short-lived and very reactive. Nevertheless, these findings correlate well with the total phenolic content, as determined using the Folin–Ciocalteu assay.

### 3.4. HPLC Analysis Results

The quantification of various bioactive compounds in the grape skin extracts using HPLC analysis. These results are presented in Table 2 and Figures S1–S12.

As shown in Table 2, all the extracts show high concentrations of different polyphenols, including phenolic acids and flavonoids. The observed antioxidant activity likely originates from the presence of these compounds, as discussed in the following section.

The highest concentration of epicatechin was observed in PSE0 (54.15 mg/g). This compound has been extensively studied for its cardiovascular, neuroprotective, metabolic, and muscle-enhancing properties. The highest concentration was recorded for PSE0, followed by PSE3, while the lowest values were determined in PSE1 and PSE2. These results align well with the polyphenolic profile of other red grape skin extracts described in the literature [3]. Naringin was the second most abundant bioactive compound in PSE0, with a 38.28 mg/g concentration. Known for its potent antioxidant properties, naringin neutralizes free radicals and exhibits anti-inflammatory effects by reducing pro-inflammatory cytokines. Additionally, it possesses antimicrobial, antiviral, neuroprotective, and anticancer properties. However, its concentration was significantly lower in PSE3 (2.66 mg/g), PSE2 (1.21 mg/g), and PSE1 (0.87 mg/g). The concentrations of caffeic acid and myricetin in PSE0 were similar, 10.51 and 9.08 mg/g, respectively. Both compounds have several hydroxyl groups attached to aromatic rings, a common structural feature characteristic of good antioxidants. Although myricetin contains five phenyl OH groups—compared to caffeic acid with only two—it should be kept in mind that the size of the radical scavenger also influences its ability to neutralize free radicals, especially in the case of bulky and sterically hindered radicals (DPPH and ABTS). The concentrations of both compounds were lower in the other extracts, with the exception of PSE3, which contained a relatively high amount of myricetin (5 mg/g).

PSE0 also contained several other bioactive compounds in significant amounts, such as apigenin (7.07 mg/g), gallic acid (5.66 mg/g), morin (4.58 mg/g), sinapic acid (4.73 mg/g), chlorogenic acid (3.02 mg/g), rutin (3.30 mg/g), and quercetin (2.32 mg/g). These compounds exhibit significant biological activity and are frequently used in biomedical formulations. In addition to the compounds discussed in the previous paragraph, these compounds also make an important contribution to the antioxidant potential of the extract. The concentrations of these compounds were moderately high in PSE3, especially quercetin, sinapic acid, and gallic acid. It should be noted that gallic acid was not found in measurable quantities in PSE1 and PSE2, while the rest of the compounds were in concentrations below or around 1 mg/g (Table 2).

Moderate concentrations of syringic acid, p-coumaric acid, ferulic acid, naringenin, and crystin—around 1 mg/g—were quantified in PSE0. The amount of syringic acid was higher in PSE3 than in PSE0 (2.81 vs. 1.19 mg/g). However, these compounds were also found in other extracts at significantly lower concentrations. It is important to note that 3,4-dihydroxybenzoic acid was found exclusively in PSE3, while it was not present in measurable quantities in the other extracts.

To analyze and compare the concentrations of polyphenolic compounds across multiple extracts (PSE0–PSE3), one-way ANOVA and post hoc analysis (Tukey’s HSD) were performed. The overall model demonstrated statistical significance (*p* = 0.005). The analysis revealed a significant effect of the extracts (PSE0–PSE3) on the concentration of polyphenolic compounds (F(3,68) = 4.759, *p* = 0.005), indicating that different extracts have significantly different mean concentrations (Table S2). Tukey’s HSD test identified significant differences in mean concentration between specific extracts, denoted by an asterisk (*) next to the mean difference values (Table S3). PSE0 exhibited significantly higher concentrations than PSE1, with a mean difference of 787.500 (*p* = 0.009), and PSE2, with a mean difference of 787.556 (*p* = 0.009). No significant difference was observed between PSE0 and PSE3 (mean difference of 610.833, *p* = 0.066). PSE1, PSE2, and PSE3 comparisons showed no significant differences (*p* > 0.05 for all pairwise comparisons). Overall, PSE0 consistently showed higher concentrations of polyphenolic compounds compared to PSE1 and PSE2, suggesting that PSE0 may possess unique properties or factors contributing to its elevated polyphenolic content. Further investigation into the source, extraction methods, and phytochemical composition of PSE0 is warranted to better understand these findings.

The findings from the HPLC analysis offer an enhanced understanding of the extracts’ compositions, highlighting the varied and plentiful presence of phenolic acids and flavonoids recognized for their bioactive properties. Significant amounts of various compounds—including quercetin, epicatechin, naringin, gallic acid, and chlorogenic acid—were identified, especially in the most active extracts (PSE0 and PSE3). The literature extensively documents these molecules, highlighting their significant antioxidant and anti-inflammatory properties, encompassing radical scavenging, metal chelation, and enzyme inhibition (e.g., LOX). The elevated levels of these compounds exhibit a significant correlation with the findings derived from the antioxidant assays, including DPPH, ABTS, FRAP, and EPR. The integration of chemical profiling with biological testing supports the assertion that the bioactivities observed are directly associated with the polyphenolic content present in the extracts.

### 3.5. LOX Inhibition Activity

The anti-LOX activity of the extracts was evaluated, revealing a wide range of inhibitory potentials. The strongest activity was obtained for PSE0 (7.9 µg/mL), followed by PSE3 (64.7 µg/mL). It is important to emphasize that PSE0 exhibits several times greater activity than the standard compound quercetin (43.2 µg/mL), while showing slightly lower activity than the standard compound NDGA (5.2 µg/mL). In contrast, PSE1 and PSE2 showed considerably weaker inhibition, with PSE1 having an IC_50_ value of 354.9 µg/mL, while the IC_50_ for PSE2 could not be determined using the applied methodology (>500 µg/mL). These findings suggest that PSE0 and PSE3 may be applied in functional foods, given their potent antioxidant and anti-inflammatory properties.

### 3.6. Antimicrobial Activity

The antimicrobial activity of extracts PSE1, PSE2, and PSE3 was evaluated across a concentration range of <0.156 to 20 mg/mL. PSE0 was tested within a concentration range of <0.39 µL/mL to 50 µL/mL. The susceptibility of various bacterial and fungal strains, including standard ATCC strains and clinical isolates, was assessed to determine the efficacy of the extracts and PSE0. The results are shown in Table 3.

Extract PSE1 exhibited significant antibacterial activity, particularly against Gram-negative bacteria. It demonstrated notable efficacy against *P. mirabilis* ATCC 12453, with a minimum inhibitory concentration (MIC) of 0.625 mg/mL. Additionally, PSE1 was active against *P. mirabilis* (MIC: 2.5 mg/mL), *P. aeruginosa*, and *S. aureus* (both isolated and ATCC strains), as well as *E. coli and B. subtilis* ATCC 6633 (MIC: 5 mg/mL). However, the *B. subtilis* isolates exhibited resistance to PSE1 at concentrations exceeding 20 mg/mL.

Extract PSE2 displayed antimicrobial activity against standard ATCC strains, particularly against Gram-negative bacteria. The strongest effect for PSE2 was shown on *P. mirabilis*—both the isolate and the ATCC strain (MIC and MBC at 5 mg/mL). Notably, the activity of PSE2 was strain dependent, demonstrating variable efficacy across different bacterial species.

Extract PSE3 exhibited the highest activity among the tested extracts. It was particularly effective against *P. mirabilis* ATCC 12453, with an MIC and MBC of ˂0.156 mg/mL. It also inhibited the growth of *P. mirabilis* at 0.625 mg/mL and *P. aeruginosa* at 2.5 mg/mL. Furthermore, *B. subtilis* ATCC 6633 showed greater susceptibility to PSE3 (MIC and MBC at 2.5 mg/mL) compared to the clinical isolate, with inhibition observed at the highest tested concentration (20 mg/mL).

The tested fungi exhibited low sensitivity to the extracts. *C. albicans* ATCC 10231 and *C. albicans* showed resistance to all the tested extracts.

The PSE0 exhibited superior antimicrobial activity compared to extracts PSE1–PSE3. The highest susceptibility was observed in *P. mirabilis* ATCC 12453, with an MIC of <0.39 µL/mL. In contrast, *E. coli* demonstrated resistance to PSE0 (>50 µL/mL). PSE0 also exhibited activity against *S. aureus* ATCC 25923 (1.56 µL/mL) and *S. aureus* clinical isolates (0.78 µL/mL). The antimicrobial effects of PSE0 were strain dependent, with *C. albicans* showing general resistance (>50 µL/mL), except for *C. albicans* ATCC 10231, which was susceptible at the highest tested concentration (50 µL/mL).

Prokupac is one of this region’s most prominent autochthonous grape varieties [47,48]. Previous studies have reported that grape seeds from the Prokupac variety possess a distinct fatty acid and soluble sugar profile [49] and exhibit the highest total phenolic content compared to seeds of other red and white grape varieties [21]. Traditionally produced red wines from autochthonous grapevine varieties have been recognized as rich sources of biologically active compounds with significant antioxidant and antimicrobial potentials [33]. In their study, *S. aureus* and *S. aureus* ATCC 25923 were identified as the most susceptible Gram-positive bacteria, while *P. mirabilis* exhibited the highest sensitivity among Gram-negative bacteria, consistent with the results of the present study. Additionally, Čomić et al. (2020) [47] reported that both *C. albicans* ATCC 10231 and a *C. albicans* clinical isolate demonstrated resistance to both dealcoholized and original wine samples, corroborating our findings.

In the present study, extracts PSE3 and PSE1 exhibited the most potent antibacterial activity. These extracts also demonstrated the highest antioxidant activity, suggesting a potential correlation between antioxidant and antibacterial properties. Previous screening studies by [50] indicated that alcohol-free red wine extracts contain bioactive compounds responsible for antimicrobial effects against Gram-positive and Gram-negative bacteria and yeasts. Furthermore, phenolic composition analysis of the tested wine extracts suggested that certain phenolic acids are likely the primary active components responsible for inhibiting pathogen growth [50]. Overall, the findings from this investigation reinforce the potential of Prokupac grape-derived extracts as rich sources of bioactive compounds with antimicrobial properties, supporting their integration into functional food systems.

## 4. Conclusions

The study demonstrates that ultrasound-assisted extracts from Prokupac grape skins—particularly PSE0 (direct extraction with 50% ethanol) and PSE3 (ethyl acetate from wine by-products)—possess significant antioxidant, anti-inflammatory, and antimicrobial properties, making them promising candidates for functional food development. PSE0 stands out with the highest content of phenolic compounds and exceptional performance across antioxidant and anti-inflammatory assays, while PSE3 excels in scavenging biologically relevant radicals and offers strong antimicrobial activity. The use of ultrasound-assisted extraction enhances the efficiency of obtaining these bioactive compounds, and the choice of solvent—50% ethanol for PSE0 and ethyl acetate for PSE3—plays a critical role in maximizing their potency.

These extracts could be incorporated into food products to provide health benefits such as protection against oxidative stress, inflammation, and certain bacterial infections. However, their limited efficacy against fungi suggests the need for targeted applications. Additionally, while PSE0 offers superior bioactivity, its direct extraction from grape skins may be less sustainable than using wine by-products (as in PSE3), an aspect that warrants further exploration. Future research should focus on integrating these extracts into food matrices and conducting in vivo studies to validate their efficacy and bioavailability, paving the way for practical functional food innovations leveraging the rich bioactive profile of Prokupac grape skins from the Šumadija region.

These findings underscore the significance of comprehensive phytochemical characterization as a basis for understanding and predicting the functional potential of preparations derived from grape skins.

## Figures and Tables

**Table 1:** Yield, antioxidant and anti-inflammatory activities, and total phenolic content of investigated grape skin extracts and standards.

Investigated Extract and Standards	Yield (%)	DPPH SC_50_ (µg/mL)	ABTS SC_50_ (µg/mL)	FRAP (A_700nm_)	TPC (mg GAE/g GSE)	LOX IC_50_ (µg/mL)
PSE1	8.9	41.9 ± 0.8	32.5 ± 0.6	0.0744 ± 0.0035	3.6 ± 0.1	354.9 ± 0.3
PSE2	6.3	99.4 ± 0.4	60.7 ± 0.3	0.0579 ± 0.0049	2.2 ± 0.1	>500
PSE3	4.9	8.3 ± 0.2	8.8 ± 0.4	0.1636 ± 0.0052	9.8 ± 0.5	64.7 ± 0.1
PSE0	9.8	0.3 ± 0.1	0.3 ± 0.1	0.2624 ± 0.0039	22.4 ± 0.1	7.9 ± 0.1
Trolox	NA	ND	1.3 ± 0.1	ND	ND	ND
NDGA	NA	0.5 ± 0.1	ND	ND	ND	5.2 ± 0.1
Quercetin	NA	0.6 ± 0.1	ND	ND	ND	43.2 ± 0.5
Ascorbic acid	NA	18.0 ± 0.1	31.1 ± 0.1	0.1249 *±* 0.0022	ND	ND

Prokupac grape skin extract in 50% ethanol (PSE1), Prokupac grape skin extract in absolute ethanol (PSE2), Prokupac grape skin extract in ethyl acetate (PSE3), and Prokupac grape skin extract without prior maceration (PSE0); Abbreviation—mg GAE/g GSE—milligrams of gallic acid equivalents (GAE) per gram of dry Grape Skin Extract; Not Determined—ND; Not Applicable—NA.

**Table 2:** Phenolic acid and flavonoid contents (mg × 10^−2^/g ± SD) in grape samples, as discussed in the following section.

	Sample	PSE0	PSE1	PSE2	PSE3
Compound					
Gallic acid		566 ± 6	N/A	N/A	470 ± 6
3,4-DHB **		N/A ***	N/A	N/A	9 ± 1
3,5-DHB		N/A	N/A	N/A	N/A
Chorogenic acid		302 ± 3	23 ± 1	32 ± 1	315 ± 9
Caffeic acid		1051 ± 26	10 ± 1	14 ± 1	31 ± 9
Syringic acid		119 ± 1	25 ± 1	29 ± 1	281 ± 6
Epicatechin		5415 ± 39	34 ± 1	42 ± 1	440 ± 9
p-Coumaric acid		126 ± 5	9 ± 1	15 ± 1	28 ± 1
Ferulic acid		142 ± 1	18 ± 14	68 ± 1	93 ± 6
Sinapic acid		473 ± 3	1 ± 1	37 ± 1	420 ± 5
Rutin		330 ± 3	190 ± 1	21 ± 1	114 ± 7
Naringin		3828 ± 54	87 ± 1	121 ± 5	266 ± 9
Myricetin		908 ± 12	29 ± 1	44 ± 1	500 ± 5
Morin		458 ± 14	96 ± 3	101 ± 1	62 ± 8
Quercetin		232 ± 9	18 ± 1	18 ± 1	211 ± 5
Naringenin		104 ± 11	85 ± 3	92 ± 3	71 ± 3
Apigenin		707 ± 5	16 ± 1	15 ± 1	514 ± 4
Crysin		77 ± 1	22 ± 1	13 ± 1	18 ± 5

** DHB—Dihydroxy benzoic acid, *** N/A—Not available.

**Table 3:** Antimicrobial activity of tested extracts.

Microorganisms	PSE1 ^1^		PSE2 ^1^		PSE3 ^1^		PSE0 ^2^		Doxycycline/Itraconazole ^1^	
	MIC	MMC	MIC	MMC	MIC	MMC	MIC	MMC	MIC	MMC
*B. subtilis* ATCC 6633	5	5	10	20	2.5	2.5	12.5	25	1.953	31.25
*B. subtilis*	>20	>20	>20	>20	20	20	6.25	12.5	0.112	1.953
*S. aureus*	5	>20	10	>20	0.625	10	0.78	1.56	0.224	3.75
*S. aureus* ATCC 25923	5	>20	10	>20	>20	>20	1.56	1.56	0.45	7.81
*P. mirabilis* ATCC 12453	0.625	1.25	5	5	<0.156	<0.156	<0.39	0.78	7.81	15.63
*P. mirabilis*	2.5	2.5	5	5	0.625	0.625	3.125	3.125	15.63	62.5
*P. aeruginosa* ATCC 27853	5	20	10	20	2.5	5	6.25	6.25	62.5	125
*P. aeruginosa*	5	20	10	20	2.5	5	6.25	12.5	250	1000
*E. coli*	20	20	20	>20	10	10	>50	>50	15.63	62.5
*E. coli* ATCC 25922	5	10	10	20	0.3125	1.25	12.5	25	15.63	31.25
*C. albicans* ATCC 10231	>20	>20	>20	>20	>20	>20	50	>50	1.95	1.95
*C. albicans*	>20	>20	>20	>20	>20	>20	>50	>50	1.95	1.95

MIC—minimum inhibitory concentration; MMC—minimum microbiocidal concentration. ^1^ Data are given in mg/mL for PSE1–PSE3 and in µg/mL for antibiotics/antimicotics. ^2^ Data are given in µL/mL for PSE0.

## Data Availability

The original contributions presented in this study are included in the article/Supplementary Materials. Further inquiries can be directed to the corresponding authors.

## Supplementary Materials

The following supporting information can be downloaded at: https://www.mdpi.com/article/10.3390/antiox14060733/s1, Table S1: Comparative analysis of antioxidant capacities across extracts (PSE3 to PSE0) using One-Way ANOVA; Table S2: Comparative analysis of concentrations of polyphenolic compounds across multiple extracts (PSE0 to PSE3) using One-Way ANOVA; Table S3: Results of Tukey’s HSD Post Hoc Test for Polyphenolic Compound Concentrations Across Extracts (PSE0 to PSE3); HPLC/DAD chromatogram of standards; HPLC/DAD chromatograms of samples produced from extract of Prokupac grape variety (Figures S1–S12).
