# Supplementary material for: Evaluation of Bioactive Properties of Ultrasound-Assisted Extracts from Prokupac Grape Skins for Functional Foods
Source: Antioxidants (Basel). 2025 Jun 15;14(6):733. doi: 10.3390/antiox14060733 (PMC12189111; doi:10.3390/antiox14060733)

Supplementary material

Evaluation of Bioactive Properties of Ultrasound-Assisted Extracts from Prokupac Grape Skins for Functional Foods

Edina Avdović [1,*], Dušan Dimić [2*] Đura Nakarada [2], Dušica Simijonović [1], Sandra Jovičić Milić [1], Katarina Marković [1], Mirjana Grujović [1], Marko Antonijević [1], Andrija Ćirić [3], Dejan Milenković [1] and Zoran Marković [4]

[1] Institute for Information Technologies, University of Kragujevac, Jovana Cvijića bb, 34000 Kragujevac, Serbia; edina.avdovic@pmf.kg.ac.rs; dusica.simijonovic@pmf.kg.ac.rs; sandra.jovicic@pmf.kg.ac.rs; katarina.mladenovic@pmf.kg.ac.rs; mirjana.grujovic@pmf.kg.ac.rs; mantonijevic@uni,kg.ac.rs

[2] Faculty of Physical Chemistry, University of Belgrade, Studentski trg 12-16, 11000 Belgrade, Serbia; ddimic@ffh.bg.ac.rs; djura@ffh.bg.ac.rs

[3] Faculty of Science, University of Kragujevac, Radoja Domanovića 12, 34000 Kragujevac, Serbia; andrija.ciric@pmf.kg.ac.rs

[4] Department of Natural Sciences and Mathematics, State University of Novi Pazar, Vuka Karadžića bb, 36300 Novi Pazar, Serbia; zmarkovic@np.ac.rs;

* Correspondence: edina.avdovic@pmf.kg.ac.rs; ddimic@ffh.bg.ac.rs

Table S1. Comparative analysis of antioxidant capacities across extracts (PSE3 to PSE0) using One-Way ANOVA.

ANOVA

DPPH_ABTS_FRAP_values

	Sum of Squares	df	Mean Square	F	Sig.
Between Groups	5175.004	3	1725.001	2.291	.155
Within Groups	6024.770	8	753.096		
Total	11199.775	11			

Table S2. Comparative analysis of concentrations of polyphenolic compounds across multiple extracts (PSE0 to PSE3) using One-Way ANOVA.

ANOVA

Concentration_of_polyphenolic_compounds

	Sum of Squares	df	Mean Square	F	Sig.
Between Groups	7541816.500	3	2513938.833	4.759	.005
Within Groups	35921254.111	68	528253.737		
Total	43463070.611	71			

Table S3. Results of Tukey's HSD Post Hoc Test for Polyphenolic Compound Concentrations Across Extracts (PSE0 to PSE3).

Multiple Comparisons

Dependent Variable: Concentration_of_polyphenolic_compounds

Tukey HSD

(I) Extracts	(J) Extracts	Std. Error	Sig.	95% Confidence Interval

		Mean Difference (I-J)			Lower Bound	Upper Bound
PSE0	PSE1	787.500*	242.270	.009	149.43	1425.57
	PSE2	787.556*	242.270	.009	149.48	1425.63
	PSE3	610.833	242.270	.066	-27.24	1248.90
PSE1	PSE0	-787.500*	242.270	.009	-1425.57	-149.43
	PSE2	.056	242.270	1.000	-638.02	638.13
	PSE3	-176.667	242.270	.885	-814.74	461.40
PSE2	PSE0	-787.556*	242.270	.009	-1425.63	-149.48
	PSE1	-.056	242.270	1.000	-638.13	638.02
	PSE3	-176.722	242.270	.885	-814.79	461.35
PSE3	PSE0	-610.833	242.270	.066	-1248.90	27.24
	PSE1	176.667	242.270	.885	-461.40	814.74
	PSE2	176.722	242.270	.885	-461.35	814.79

*. The mean difference is significant at the 0.05 level.

Figure S1: Chromatogram of PSE1 at 254 nm

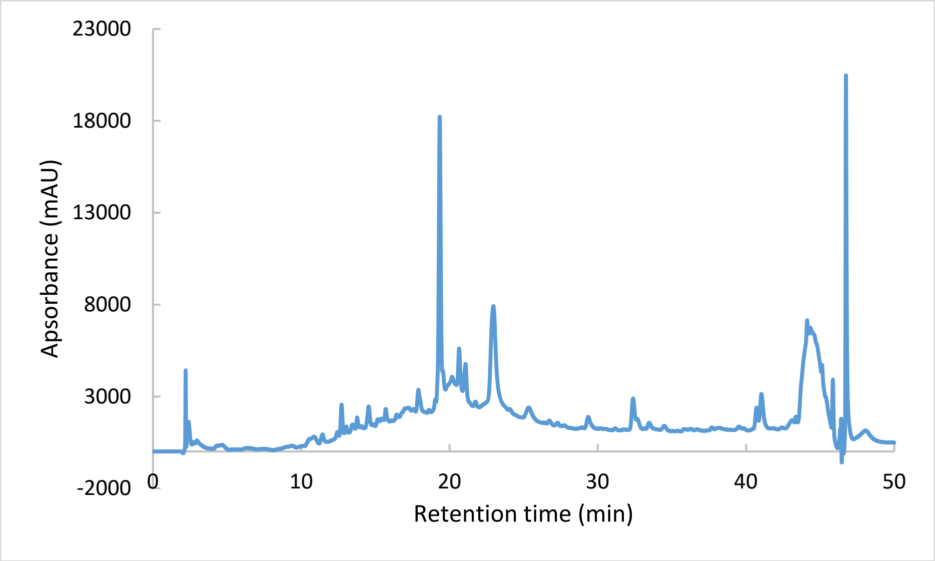

Figure S2: Chromatogram of PSE1 at 280 nm

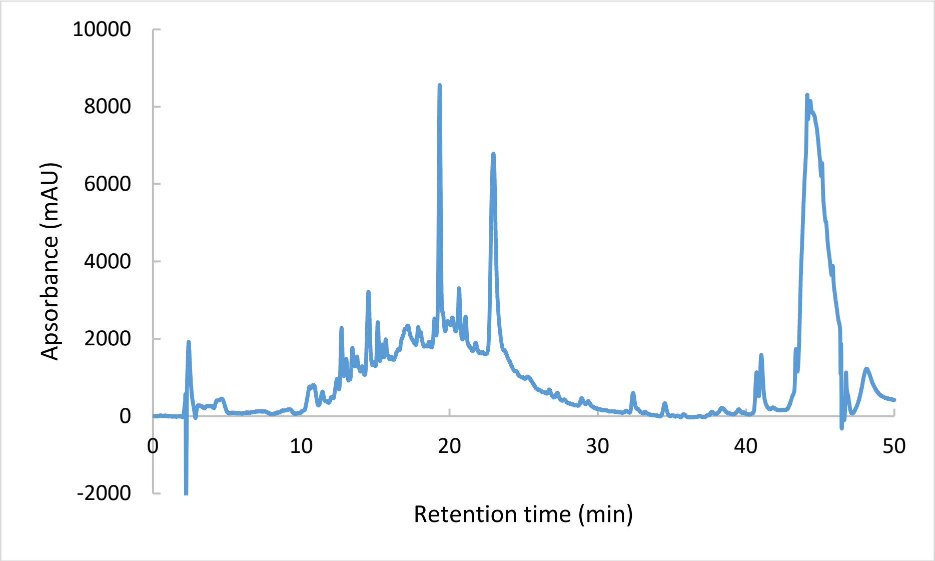

Figure S3: Chromatogram of PSE1 at 360 nm

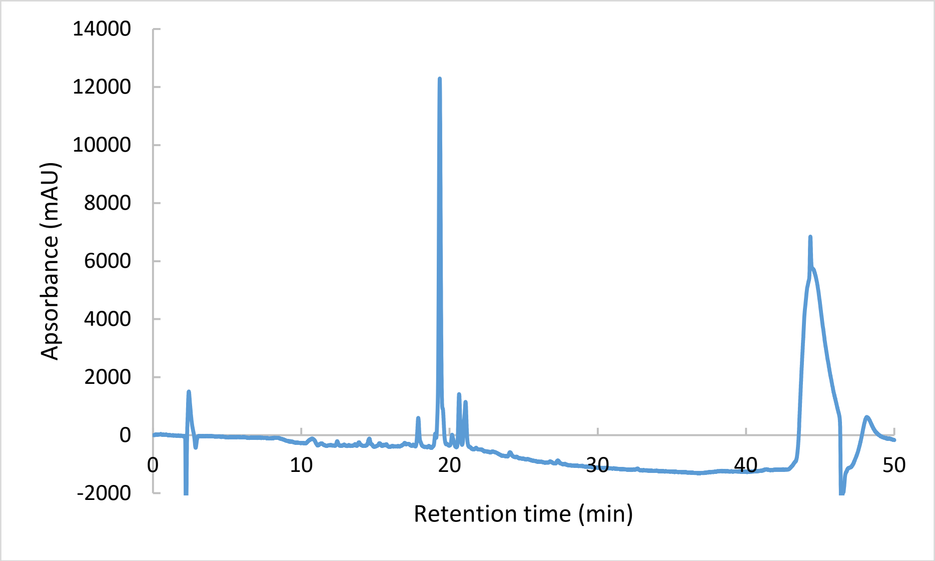

Figure S4: Chromatogram of PSE2 at 254 nm

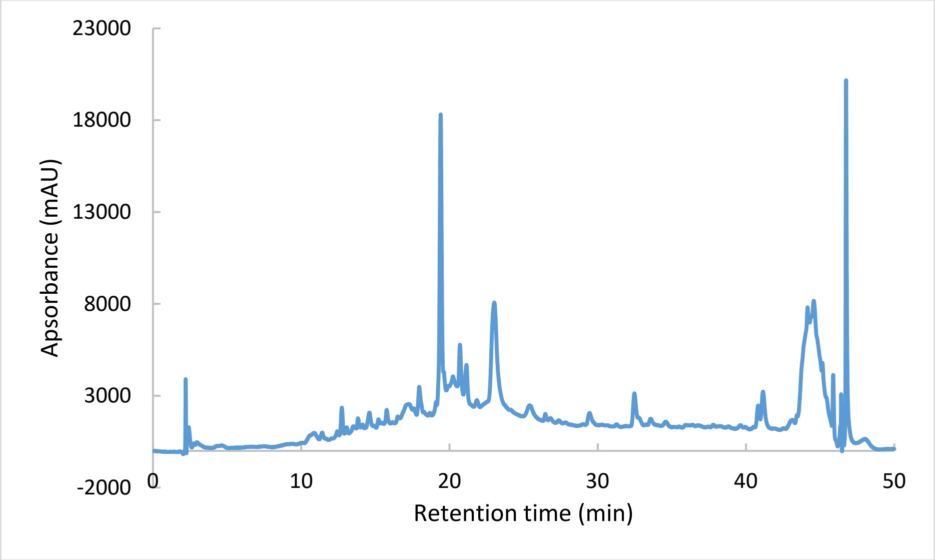

Figure S5: Chromatogram of PSE2 at 280 nm

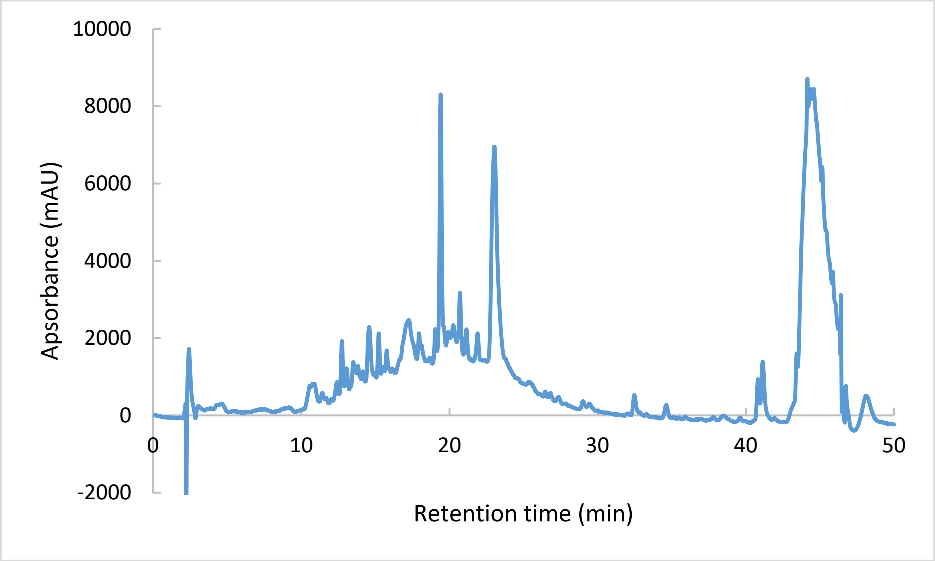

Figure S6: Chromatogram of PSE2 at 360 nm

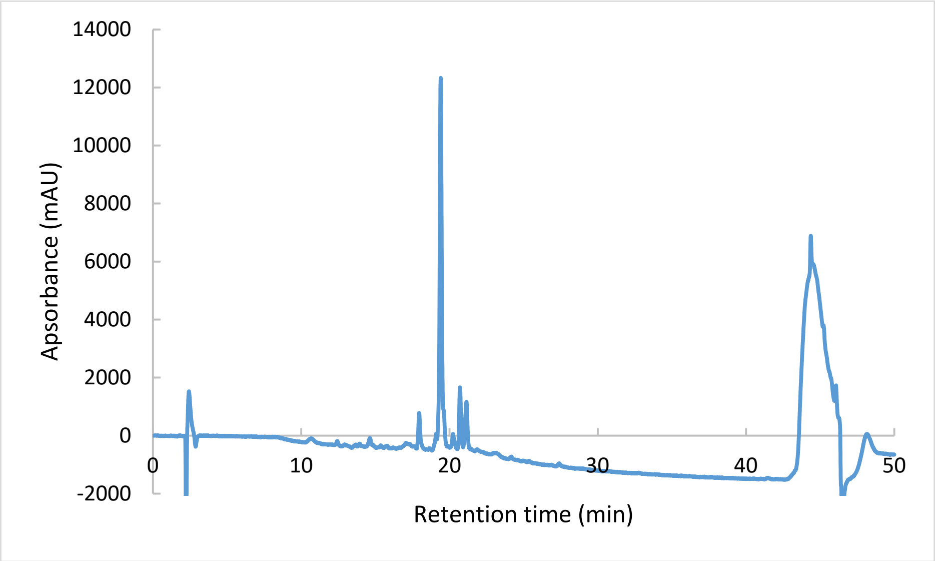

Figure S7: Chromatogram of PSE3 at 254 nm

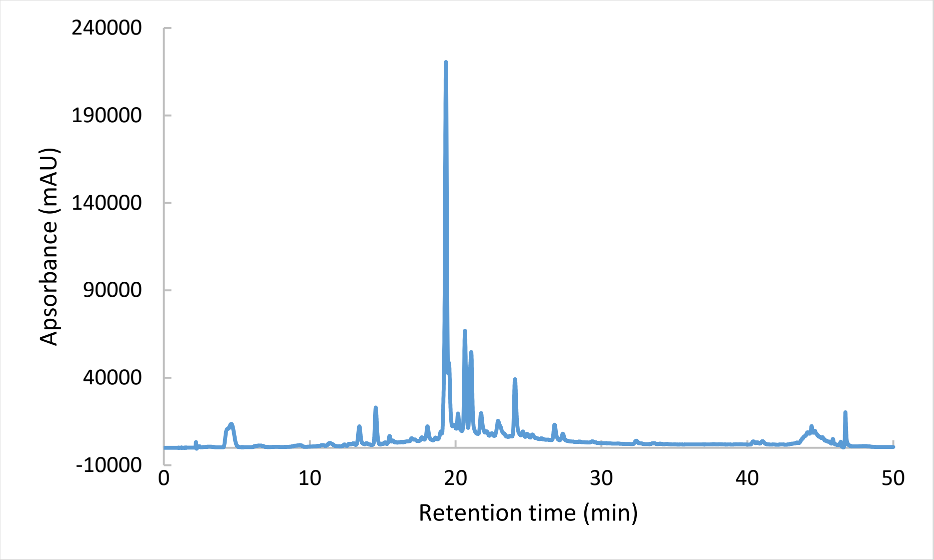

Figure S8: Chromatogram of PSE3 at 280 nm

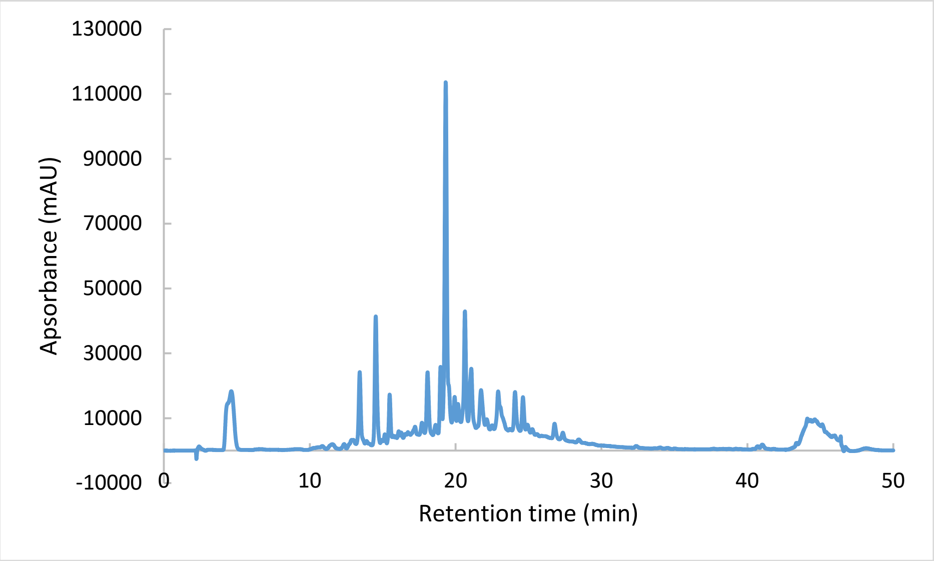

Figure S9: Chromatogram of PSE3 at 360 nm

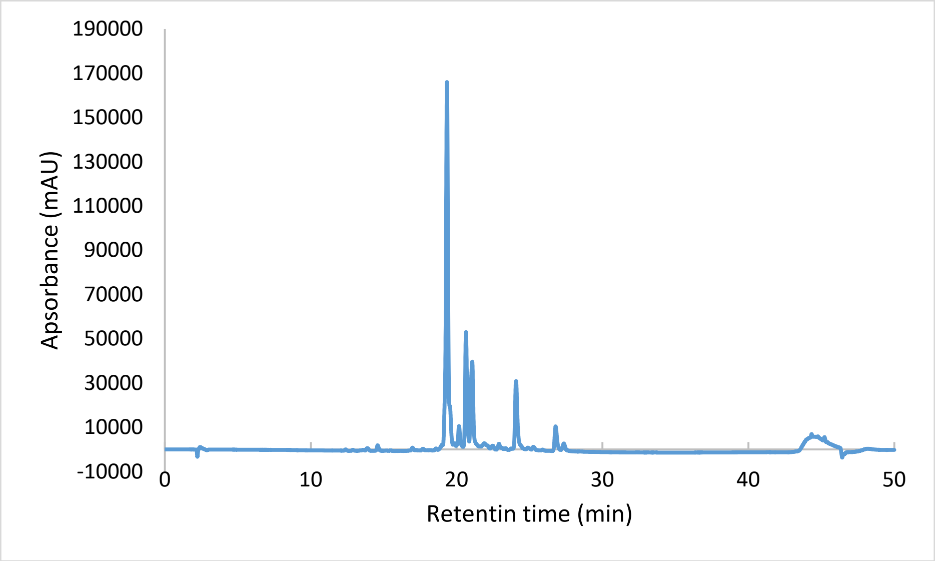

Figure S10: Chromatogram of PSE0 at 254 nm

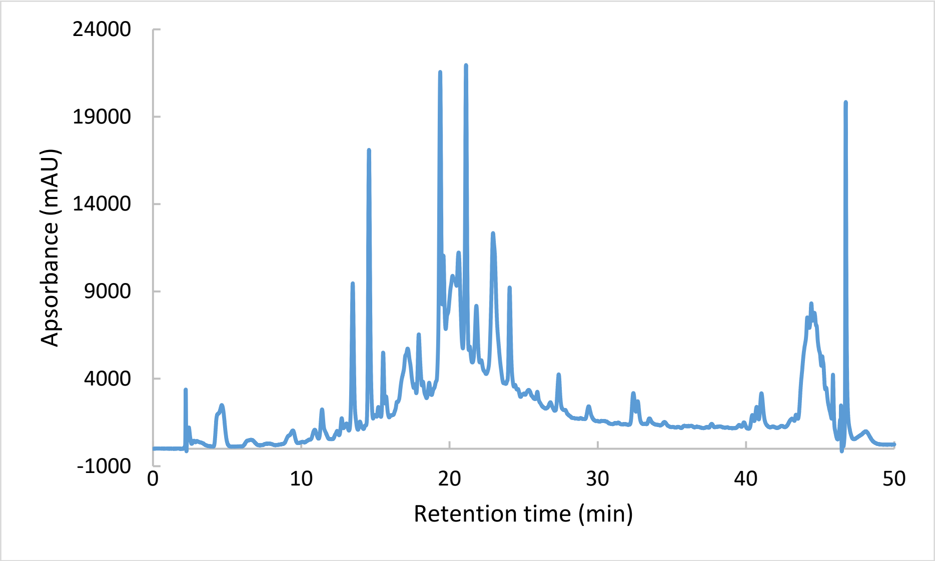

Figure S11: Chromatogram of PSE0 at 280 nm

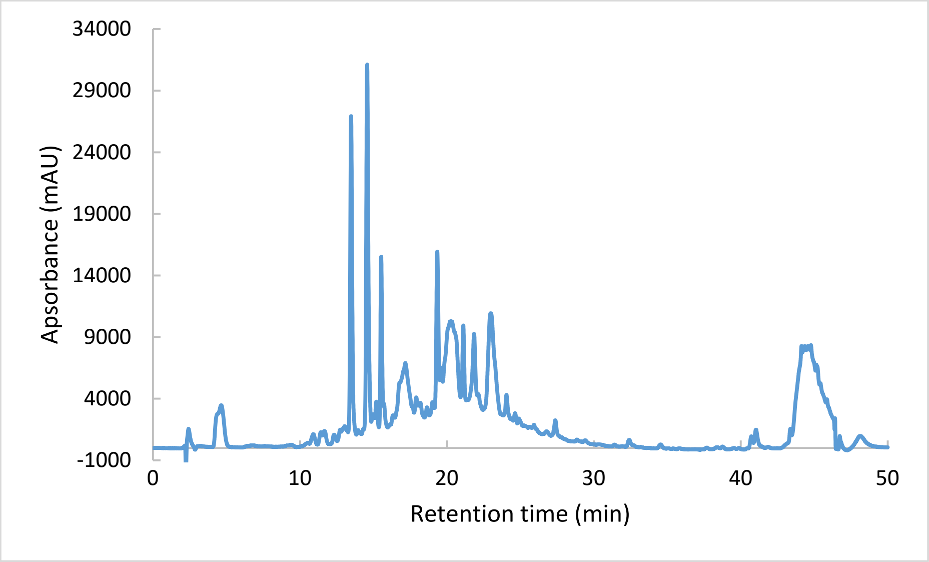

Figure S12: Chromatogram of PSE0 at 360 nm

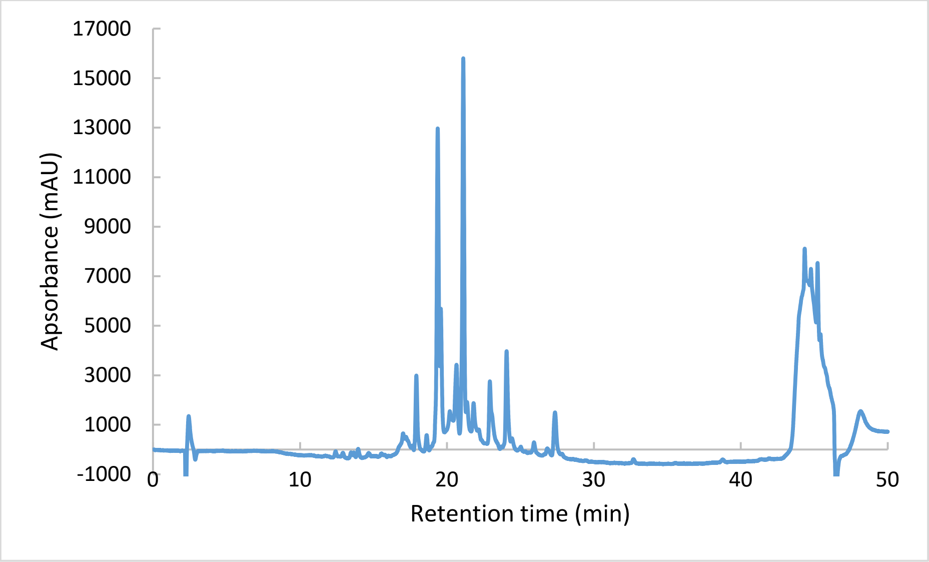

Supplement: Supplementary file 1 [file antioxidants-14-00733-s001.zip › antioxidants-3632241-supplementary.pdf]
